# Acupuncture-related peripheral stimulation as a candidate neuromodulatory model of insomnia: clinical evidence, sleep-wake circuitry, and translational limits

**DOI:** 10.3389/fnins.2026.1884710

**Published:** 2026-08-26

**Authors:** He Ming, Jie Yuan, Jiao Liang, Penglong Yu

**Affiliations:** 1 Key Laboratory of Acupuncture-Moxibustion and Tuina Intelligent Equipment of Chongqing Administration of Traditional Chinese Medicine, Chongqing Three Gorges Medical College, Chongqing, China; 2 Shaanxi Provincial Hospital of Traditional Chinese Medicine, Xi'an, Shaanxi, China

**Keywords:** acupuncture, hyperarousal, insomnia disorder, locus coeruleus, peripheral neuromodulation, sleep EEG, sleep–wake circuitry

## Abstract

Acupuncture-related peripheral stimulation is often discussed as a non-pharmacological option for insomnia disorder, but its relevance to sleep neuroscience depends on more than improvement in sleep questionnaires. This critical narrative Review evaluates whether acupuncture and related acupoint-based stimulation methods can serve as testable peripheral models of sleep–wake and hyperarousal circuitry. Across clinical studies, the most consistent signal is improvement in subjective sleep quality and insomnia severity in selected populations. Evidence for objective sleep architecture, multiday sleep–wake stability, relapse prevention, and biomarker-mediated clinical response remains less secure. Human neuroimaging studies provide emerging but still associative evidence involving locus coeruleus, hypothalamic, insular, limbic–prefrontal, and thalamocortical networks. Electrophysiological, autonomic, endocrine, neurochemical, and microbiota–gut–brain findings are biologically plausible, but their proximity to human chronic insomnia disorder varies substantially. Safety is generally favorable when appropriately delivered, although adverse-event reporting remains inconsistent and comparative cost-effectiveness against CBT-I or pharmacotherapy is unestablished. We propose that acupuncture-related stimulation should currently be evaluated as an adjunctive, preference-sensitive, and mechanistically testable approach rather than as a replacement for cognitive behavioral therapy for insomnia. Its translational credibility will depend on credible comparators, objective sleep physiology, expectancy assessment, and prespecified mediation analyses.

## 1 Introduction

Insomnia disorder is defined by persistent difficulty initiating sleep, maintaining sleep, or returning to sleep after early-morning awakening, despite adequate opportunity for sleep and with clinically meaningful daytime consequences. It is not simply a nighttime complaint. Chronic insomnia interacts with mood, cognition, autonomic tone, immune function, metabolic regulation, and quality of life. Population studies indicate that insomnia symptoms are common, whereas chronic insomnia disorder affects a smaller but clinically important proportion of adults (Morin et al., 2011; Riemann et al., 2023). Persistent sleep disturbance is also associated with fatigue, impaired concentration, anxiety and depressive symptoms, increased health-care use, and suicidal thoughts or behaviors, although these associations are strongly shaped by medical and psychiatric comorbidity (Pigeon et al., 2012).

Current guidelines place cognitive behavioral therapy for insomnia (CBT-I) as the first-line treatment for chronic insomnia disorder (Qaseem et al., 2016; Edinger et al., 2021; Riemann et al., 2023). Pharmacotherapy remains appropriate for selected patients, particularly when rapid symptom relief is required or behavioral treatment is unavailable. Guideline documents, however, also emphasize individualized risk-benefit assessment and attention to next-day sedation, falls, tolerance, dependence, and cognitive adverse effects (Sateia et al., 2017; Matheson et al., 2024). In routine care, CBT-I is not always accessible, digital CBT-I is not acceptable to every patient, and some patients prefer body-based or integrative approaches. These circumstances do not justify replacing established treatment with acupuncture. They do justify a more precise question: whether acupuncture has an adjunctive, preference-sensitive, or mechanistically informative role.

Hyperarousal refers to sustained cognitive–emotional, cortical, autonomic, and neuroendocrine activation that persists into the intended sleep period and destabilizes sleep initiation or maintenance. Its manifestations can include sleep-related rumination, elevated sympathetic or stress-axis activity, and increased high-frequency EEG activity, although individual patients need not express every component of this multidimensional phenotype (Bonnet and Arand, 1997; Perlis et al., 2001; Zhao W. et al., 2021). Relevant systems include the locus coeruleus (LC), dorsal raphe nucleus, hypothalamic sleep–wake nuclei, suprachiasmatic nucleus (SCN), thalamic reticular nucleus (TRN), salience network, default-mode network, and limbic–prefrontal circuits. These systems are regulated by GABA, glutamate, serotonin, melatonin, norepinephrine, dopamine, corticotropin-releasing hormone, cortisol, and autonomic–vagal inputs (Benarroch, 2018; Eschenko and Sara, 2008).

A neuroscience Review of acupuncture for insomnia therefore has to ask a narrower question than whether sleep scores improve. It should ask whether peripheral acupoint stimulation can measurably perturb arousal-related neural systems and whether those changes explain clinical benefit. The answer matters because acupuncture is not a single neurophysiological stimulus. Manual acupuncture, electroacupuncture, transcutaneous electrical acupoint stimulation (TEAS), auricular stimulation, and transcutaneous auricular vagus nerve stimulation (taVNS) differ in tissue penetration, receptor recruitment, stimulation parameters, practitioner interaction, and patient expectation. The phrase acupuncture-related peripheral stimulation is used in this Review when the evidence base extends beyond manual or electroacupuncture.

This Review evaluates acupuncture-related stimulation as a candidate peripheral neuromodulatory model of sleep-wake transition instability and hyperarousal networks. In this Review, the term candidate model does not imply that acupuncture-related stimulation is already a validated circuit-specific intervention. Rather, it denotes a peripheral stimulation framework through which testable hypotheses about sleep-wake regulation, hyperarousal, and clinical response can be generated. The Review has three aims. First, it separates direct chronic-insomnia evidence from comorbid and acute-state sleep-disturbance studies. Second, it appraises human neuroimaging, objective sleep physiology, autonomic-endocrine biomarkers, neurochemical findings, and animal experiments according to their proximity to human chronic insomnia disorder. Third, it identifies the evidence that would strengthen, weaken, or falsify a peripheral neuromodulation model.

### 1.1 Scope and added value of this review

Previous reviews have summarized acupuncture for broad sleep disorders, specific clinical populations, or rodent models. A recent Frontiers Review, for example, focused on acupuncture mechanisms in rodent models of sleep disorders and emphasized brain regions and neurotransmitter changes (Lee and Kim, 2023). Other systematic reviews have estimated pooled effects on sleep scales or compared different acupuncture modalities (Cheuk et al., 2012; Zhao F. Y. et al., 2021; Xu et al., 2024; Yu et al., 2025; Fang et al., 2026; Hu et al., 2026). These syntheses are useful, but they do not by themselves answer a translational neuroscience question.

The present Review takes a different route. It centers on insomnia disorder and insomnia-related clinical phenotypes, integrates human sham-controlled trials with neuroimaging and biomarker studies, and weighs preclinical mechanisms according to their translational distance from human chronic insomnia disorder. Direct clinical trials, associative human imaging, autonomic and endocrine biomarkers, serum neurotransmitter changes, and animal circuit experiments are kept in separate evidential lanes. This hierarchy is intended to reduce mechanistic overreach while preserving the conceptual value of acupuncture as a peripheral stimulation model relevant to sleep-wake circuitry.

This framing also clarifies the clinical question. Acupuncture is not considered here as a generic substitute for CBT-I. It is evaluated as a proposed peripheral neuromodulatory approach that may be useful for selected patients, particularly when insomnia coexists with pain, affective symptoms, autonomic hyperarousal, medication intolerance, or limited access to behavioral sleep medicine. The Review therefore emphasizes what the evidence can support, where it remains thin, and which future experiments would make the field more falsifiable.

## 2 Literature selection and critical appraisal framework

This critical narrative Review uses purposive source selection to evaluate acupuncture-related peripheral stimulation as a testable neuromodulation model of insomnia. The goal was conceptual and translational synthesis rather than a pooled efficacy estimate. The process was therefore documented as a narrative source-selection audit, not as PRISMA-compliant systematic screening; duplicate independent screening and formal study-level risk-of-bias grading were not undertaken.

The exact core PubMed/MEDLINE search was rerun on 7 August 2026. Earlier targeted searches of the Cochrane Library, publisher pages, reference lists, and major evidence syntheses were completed through 11 May 2026 and selectively updated during revision. Recent systematic reviews that searched CNKI, Wanfang, VIP, and SinoMed were used as evidence maps because acupuncture research is heavily represented in Chinese-language databases; they were not treated as a substitute for an independent bilingual systematic review. The absence of duplicate screening across Chinese databases, Embase, and Web of Science remains a limitation.

The exact core PubMed string was: (insomnia disorder OR chronic insomnia OR primary insomnia) AND (acupuncture OR electroacupuncture OR transcutaneous electrical acupoint stimulation OR auricular stimulation OR acupoint stimulation) AND (sham acupuncture OR polysomnography OR actigraphy OR electroencephalography OR spectral EEG OR fMRI OR functional connectivity OR locus coeruleus OR hypothalamus OR suprachiasmatic nucleus OR thalamic reticular nucleus OR HPA axis OR GABA OR glutamate OR serotonin OR melatonin OR heart rate variability OR microbiota OR gut-brain axis). A reproducibility check of this string on 7 August 2026 returned 360 PubMed records, and all 360 titles underwent a single-pass, revision-stage relevance audit. At title level, 92 records concerned direct CID clinical or human acupuncture-related evidence, 82 concerned indirect clinical populations or acute states, 122 concerned human sleep mechanisms, reviews, or guidelines, 20 concerned preclinical insomnia, and 44 were clearly irrelevant. These categories guided narrative prioritization and were not full-text eligibility decisions. Details are reported in Supplementary Table S1.

The earlier iterative searches of the Cochrane Library, publisher sites, and reference lists were not prospectively logged as unique records; their deduplicated record and full-text counts therefore cannot be reconstructed without false precision. The final cited synthesis contains 63 publications drawn from the core search and targeted supplementation. Supplementary Figure S1 shows which counts are directly auditable and which were unavailable. These numbers describe transparency of source selection rather than a systematic-review eligibility flow.

Priority was given to randomized controlled trials, sham-controlled studies, comparative effectiveness trials, clinical guidelines, systematic reviews, human neuroimaging studies, biomarker studies, and animal experiments in insomnia or sleep-disorder models with sleep-relevant endpoints. Studies in which insomnia was not the primary clinical target were used only when they clarified a pathway directly relevant to sleep-wake regulation. Evidence from pain, Parkinson disease, stroke spasticity, addiction, or other non-insomnia models was not treated as proof of an insomnia mechanism. Non-peer-reviewed sources, uncontrolled reports, and studies using only broad sleep-quality complaints without insomnia-specific outcomes were deprioritized.

The appraisal framework is conservative. Randomized or sham-controlled human trials and human imaging or physiology studies in insomnia disorder have the highest clinical proximity, although even these data rarely establish mechanism. Human endocrine, autonomic, serum neurotransmitter, and sleep-physiology measures are intermediate evidence when they accompany clinical change but have not been tested as temporal mediators. Animal and cellular work can access tissue and circuits but cannot reproduce the cognitive, social, chronicity, comorbidity, and expectancy components of human insomnia. Confidence in each inference therefore reflects design, directness to chronic insomnia disorder, objective measurement, consistency, independent replication, and causal interpretability; it is a structured narrative appraisal rather than formal GRADE.

## 3 Clinical evidence for acupuncture-related stimulation in insomnia

Clinical studies of acupuncture for insomnia are heterogeneous in ways that matter. Chronic insomnia disorder is the core condition for this Review. Comorbid insomnia, including insomnia with depression or after stroke, may extend the clinical rationale but is not interchangeable with primary chronic insomnia. Pregnancy-related insomnia has distinct safety and endocrine considerations. Cancer survivor insomnia is clinically important because CBT-I is an active benchmark rather than a wait-list comparison. Perioperative sleep disturbance is a short-term hospital-related state shaped by pain, anesthesia, inflammation, and environmental disruption. It should not be pooled conceptually with chronic insomnia disorder.

Older evidence syntheses were appropriately cautious. A Cochrane review of 33 randomized trials concluded that low methodological quality, heterogeneity, and publication bias prevented reliable conclusions about acupuncture for insomnia (Cheuk et al., 2012). More recent reviews are more favorable for subjective sleep outcomes, but they continue to note uncertainty around blinding, sham credibility, objective sleep indices, long-term maintenance, and modality-specific inference (Zhao F. Y. et al., 2021; Xu et al., 2024; Yu et al., 2025; Fang et al., 2026; Hu et al., 2026). A fair reading is that acupuncture has a clinically relevant signal in selected settings, but the field has not established a uniform stand-alone effect across all insomnia populations and objective outcomes.

The most recent practice-oriented guidance is also measured. An international evidence-based guideline conditionally suggested manual acupuncture or electroacupuncture for chronic insomnia in settings where these interventions are available, but the recommendation was based on moderate-to-low certainty evidence and multidisciplinary consensus rather than definitive mechanistic proof (Wang et al., 2026). For a neuroscience readership, this distinction is important: a conditional clinical recommendation does not demonstrate the neural route by which benefit occurs.

### 3.1 Chronic insomnia disorder

Across direct chronic-insomnia evidence, the clearest pattern is stronger improvement in PSQI and ISI than in objective or durable outcomes. Wang et al. (2021) randomized 82 adults to acupuncture at HT7 and KI7 or shallow sham needling over 10 sessions. Questionnaire outcomes and selected PSG indices—sleep-onset latency, N1 percentage, and N3 percentage—favored acupuncture immediately after treatment, but the between-group differences were not maintained at the two post-treatment follow-up assessments. The study therefore supports a short-term, outcome-dependent signal rather than durable relapse prevention or a specific sleep–wake circuit mechanism.

Recent evidence syntheses reinforce this subjective–objective boundary. A review of 10 sham-controlled trials found improvement in PSQI and ISI but uncertainty for objective total sleep time (Yu et al., 2025). Electroacupuncture meta-analysis also favored subjective sleep quality while emphasizing small, single-center studies and variable quality (Xu et al., 2024). A network meta-analysis reported short-term advantages for several modalities but no robust superiority among acupuncture modalities and limited safety data (Fang et al., 2026). Together with the objective-outcome review by Zhao F. Y. et al. (2021), these syntheses support a recurrent short-term subjective signal; total sleep time, sleep efficiency, wake after sleep onset, and long-term maintenance remain less secure.

Human imaging narrows the hypotheses but does not yet provide independent causal replication. Chen et al. (2023) and Jiang et al. (2024) reported LC and emotion-network findings from different analyses of the same registered trial (ChiCTR1800015282), whereas Peng et al. (2024) examined a separate hypothalamic-connectivity cohort. Across these reports, connectivity change accompanied or was associated with symptom change, but small analyzed samples, region-of-interest and multiplicity choices, attrition, and the absence of prospective mediation prevent identification of a treatment-specific neural mechanism. Their value is mechanistic proximity and hypothesis refinement, not proof of circuit normalization.

### 3.2 Insomnia with psychiatric, neurological, or cancer-related comorbidity

In insomnia with depression, Yin et al. (2022) conducted a multicenter randomized clinical trial of 270 participants and compared electroacupuncture plus standard care, sham acupuncture plus standard care, and standard care alone. Electroacupuncture produced larger improvements in PSQI and ISI scores, with effects observed through follow-up and no serious adverse events reported. Liu et al. (2021) also examined acupuncture for anxiety and depression symptoms in chronic insomnia, supporting the clinical relevance of affective arousal as a treatment target. These findings should not be generalized uncritically to primary insomnia because depressive symptoms, antidepressant use, stress physiology, and expectancy may influence both sleep and treatment response.

Post-stroke insomnia provides another clinically relevant but mechanistically distinct population. In an assessor- and participant-blinded trial, Cao et al. (2022) assigned 144 patients with ischemic stroke and insomnia to verum or sham acupuncture three times weekly for 4 weeks. Verum acupuncture improved insomnia severity and actigraphic sleep efficiency during treatment and follow-up, and adverse events were mild. Systematic reviews of post-stroke insomnia also tend to favor acupuncture, although comparator choice, concomitant therapy, and neurological heterogeneity limit interpretation (Yang, 2021; Su et al., 2024). Stroke-related network injury, motor disability, fatigue, medication use, and rehabilitation context make this population indirect for chronic insomnia disorder mechanisms.

For cancer survivors, Garland et al. (2019) compared acupuncture with CBT-I in 160 survivors with insomnia. Both interventions produced durable improvement, but CBT-I was superior for insomnia severity immediately after treatment. Acupuncture showed potential advantages for pain in exploratory analyses (Yang et al., 2021). This trial is clinically important because it prevents overstatement: acupuncture may be useful within personalized survivorship care, especially when pain and other somatic symptoms are prominent, but the evidence does not show equivalence to CBT-I for insomnia severity. A recent clinical review similarly framed acupuncture as a potential adjunct to CBT-I rather than a replacement for it (Kutana et al., 2023).

### 3.3 Pregnancy-related and perioperative sleep disturbance: indirect evidence

Pregnancy-related insomnia and perioperative sleep disturbance require separate interpretation. Foroughinia et al. (2020) reported that acupuncture improved PSQI scores and increased urinary 6-sulfatoxymelatonin in pregnant women, but attrition and the endocrine and safety context of pregnancy limit extrapolation. Trials of TEAS after surgery suggest improved postoperative sleep quality and pain outcomes (Song et al., 2020; Wang et al., 2023). These findings are promising for acute care, but perioperative sleep disruption is not the same clinical entity as chronic insomnia disorder. They are best read as boundary-condition evidence rather than direct support for a chronic-insomnia neuromodulation model.

### 3.4 Daytime functioning and patient-centered outcomes

Insomnia disorder is diagnosed partly by daytime impairment, yet acupuncture trials often treat daytime function as a secondary or exploratory outcome. This limits the clinical and mechanistic interpretability of the literature. If peripheral stimulation reduces hyperarousal, clinically meaningful effects should extend beyond sleep-onset latency and PSQI scores to fatigue, vigilance, affective arousal, cognitive performance, and daily functioning. A recent meta-analysis focused on daytime dysfunction reported favorable effects of acupuncture on insomnia symptoms and several daytime indices, while also emphasizing variability in measures and the need for better trial quality (Li et al., 2026). Daytime improvement is clinically important, but it becomes mechanistically informative only when linked prospectively to objective sleep physiology, arousal markers, or prespecified neural and autonomic mediators.

### 3.5 Summary of clinical certainty

The clinical signal is clearer than it was a decade ago, but it remains outcome-dependent. Support is strongest for short-term subjective sleep quality and insomnia severity, whereas objective sleep architecture, multiday sleep–wake patterns, relapse prevention, and biomarker-mediated response remain less settled. The defensible clinical position is evaluation as an adjunctive or preference-sensitive intervention in selected settings, not replacement of CBT-I. Table 1 therefore distinguishes the confidence warranted for each specific clinical or mechanistic inference rather than assigning one global evidence grade to acupuncture.

**Table 1:** Key clinical and mechanistic evidence relevant to chronic insomnia disorder.

Study/evidence	Population and design	Comparator and outcomes	Objective or biomarker measures	Interpretation for CID and mechanism	Narrative confidence for stated inference
Cheuk et al. (2012)	Cochrane review; 33 randomized trials; diverse acupuncture modalities and insomnia populations.	Possible benefit in some comparisons, but low quality, heterogeneity, and publication bias prevented reliable conclusions.	Objective outcomes inconsistently reported.	Historical benchmark; not evidence of mechanism.	Very low for efficacy; no mechanistic inference.
Zhao F. Y. et al. (2021)	Systematic review and meta-analysis focused on objective sleep indices in primary insomnia.	Subjective measures generally favored acupuncture, but objective results were less consistent.	PSG or actigraphy outcomes reviewed.	Highlights the subjective-objective discrepancy; mechanism cannot be inferred from scores alone.	Low for objective benefit; very low for mechanism.
Yu et al. (2025); Fang et al. (2026)	Recent sham-controlled and network meta-analytic evidence in chronic or primary insomnia.	Subjective PSQI/ISI outcomes generally favored acupuncture-related interventions, but comparative modality superiority remained uncertain.	Objective sleep and safety data remained limited or heterogeneous.	Directly relevant to CID; supports continued evaluation but not causal neuromodulation.	Low–moderate for short-term subjective benefit; very low for mechanism or durability.
Hu et al. (2026)	Systematic review and meta-analysis of sleep and endocrine outcomes.	Favorable signals for PSQI and selected sleep outcomes, with substantial heterogeneity.	Serum melatonin and salivary cortisol signals reported; salivary melatonin inconsistent.	Endocrine findings are proposed intermediates, not proof of mediation.	Low for endocrine association; very low for mediation.
Li et al. (2026)	Meta-analysis focusing on daytime dysfunction in insomnia.	Reported improvement in insomnia symptoms and several daytime-function indices.	Daytime outcomes varied by measure.	Important patient-centered evidence; requires objective sleep and mediator linkage.	Low for daytime benefit; very low for mechanism.
Wang et al. (2026)	International evidence-based guideline for insomnia management.	Conditionally suggests manual acupuncture or electroacupuncture where available.	Not a mechanistic study.	Useful for clinical positioning; recommendation certainty is moderate-to-low.	Low–moderate for a conditional clinical recommendation; not mechanistic evidence.
Wang et al. (2021)	Randomized single-blind sham-controlled trial; 82 adults with CID.	Acupuncture at HT7 and KI7 over 10 sessions vs. shallow sham needling; PSQI and ISI reductions favored acupuncture.	PSG suggested shorter sleep-onset latency, lower N1 percentage, and higher N3 percentage.	Direct short-term evidence; between-group differences were not maintained at two follow-ups, limiting durability and causal inference.	Moderate for immediate subjective and selected PSG outcomes; very low for durability or mechanism.
Chen et al. (2023)	Randomized sham-controlled fMRI trial; 60 recruited and 50 analyzed with insomnia disorder.	Real acupuncture vs. superficial sham; PSQI change favored real acupuncture (−4.73 ± 3.47 vs. −1.25 ± 2.56).	Actigraphy included; LC functional connectivity was the main mechanistic endpoint.	Direct human association, but this is one analysis of ChiCTR1800015282; small sample and no mediation or independent replication.	Low for a connectivity association; very low for mediation or independent replication.
Yin et al. (2022)	Multicenter RCT; 270 patients with insomnia comorbid with depression.	Electroacupuncture plus standard care vs. sham plus standard care and standard care alone.	Actigraphy showed improvement in total sleep time; follow-up extended to week 32.	Moderate relevance; affective arousal and antidepressant context complicate sleep-specific attribution.	Moderate for comorbid clinical benefit; low directness to CID mechanism.
Garland et al. (2019)	Comparative effectiveness RCT; 160 cancer survivors with insomnia.	Acupuncture vs. CBT-I; both improved, but CBT-I produced larger immediate ISI reduction.	Objective sleep was not the central mechanistic endpoint.	Important active-comparator boundary evidence; acupuncture should not be described as equivalent to CBT-I for insomnia severity.	Moderate for population-specific comparative effectiveness; very low for mechanism.

Confidence ratings are a structured narrative appraisal of the specific inference shown, based on study design, directness to CID, objective measurement, consistency, replication, and causal interpretability; they are not formal GRADE ratings. A study may therefore warrant higher confidence for short-term subjective clinical benefit but very low confidence for a neural mechanism. CBT-I, cognitive behavioral therapy for insomnia; CID, chronic insomnia disorder; fMRI, functional magnetic resonance imaging; ISI, insomnia severity index; LC, locus coeruleus; MD, mean difference; PSG, polysomnography; PSQI, Pittsburgh sleep quality index; RCT, randomized controlled trial; SE, sleep efficiency; TEAS, transcutaneous electrical acupoint stimulation; TST, total sleep time; WASO, wake after sleep onset; WMD, weighted mean difference.

## 4 Neurobiological mechanisms

Acupuncture is unlikely to act through a single sleep-promoting mechanism. Peripheral stimulation at acupoints may activate somatosensory, connective-tissue, vascular, immune, and autonomic afferent pathways. These inputs can interact with spinal, brainstem, hypothalamic, limbic, thalamic, and cortical systems that participate in sleep-wake regulation. The challenge is to distinguish plausible biological pathways from demonstrated mechanisms. A defensible mechanistic claim should ideally trace a causal chain from peripheral stimulation, to afferent signaling, to a measurable neural or physiological mediator, and then to objective sleep or clinical improvement.

**Figure 1:**
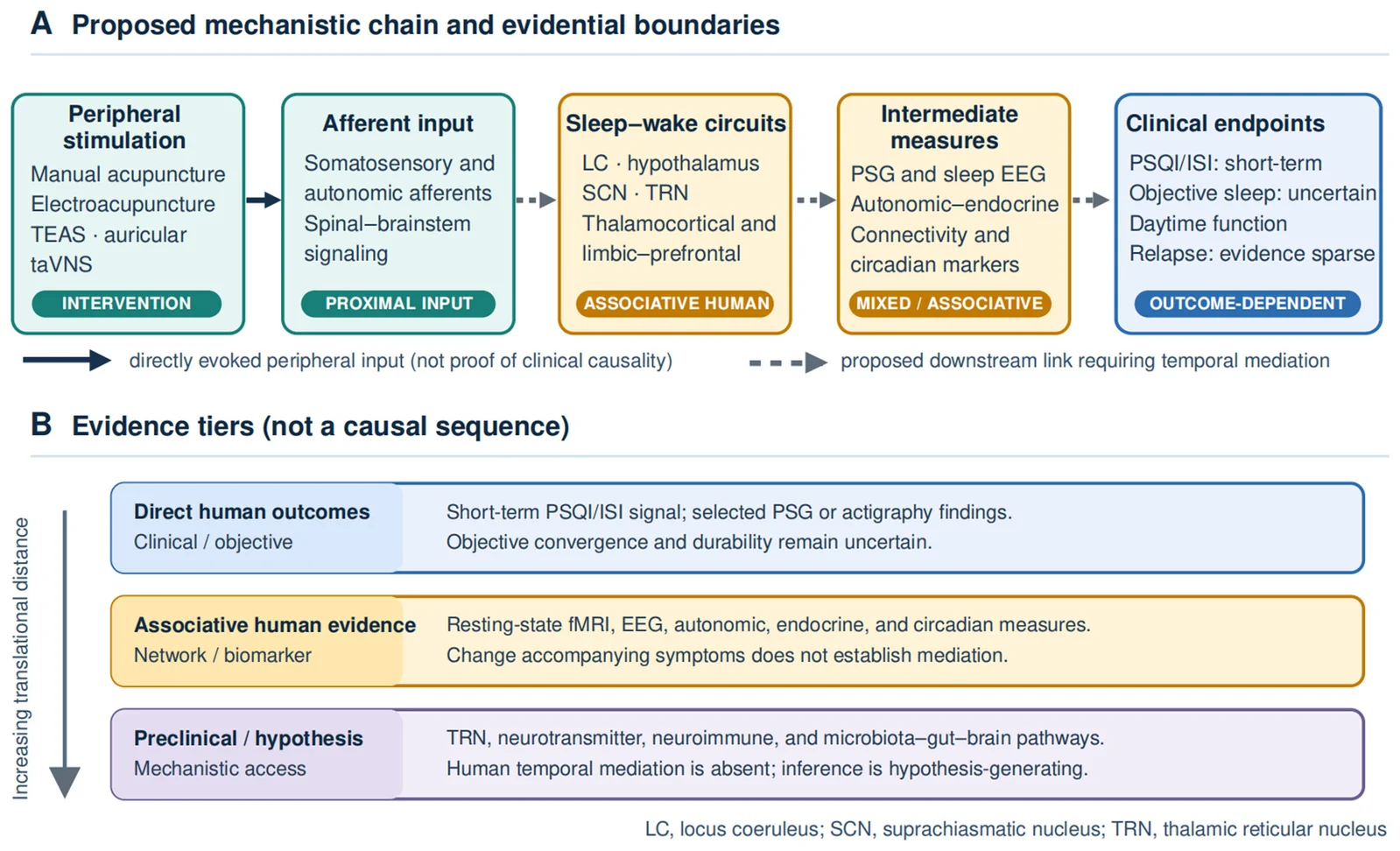
Proposed framework of acupuncture-related peripheral stimulation as a neuromodulatory model of insomnia. **(A)** Proposed chain from peripheral stimulation through afferent input, sleep–wake circuits, intermediate measures, and clinical endpoints. The solid arrow denotes the directly evoked peripheral afferent input; it does not establish clinical causality. Dashed arrows denote downstream circuit, intermediate-measure, and clinical links that remain hypothetical and require temporal and mediation testing. **(B)** Evidence tiers distinguish direct human clinical or objective evidence, associative human network or biomarker evidence, and preclinical or hypothesis-generating evidence without implying causal progression. Evidence of symptom improvement does not establish the complete chain shown. LC, locus coeruleus; SCN, suprachiasmatic nucleus; TEAS, transcutaneous electrical acupoint stimulation; TRN, thalamic reticular nucleus.

This distinction is particularly important in acupuncture research. Human fMRI and endocrine studies show associations with symptom change but usually cannot establish causality. Serum neurotransmitter levels do not necessarily reflect central synaptic transmission. Rodent studies can probe tissue and circuits, but they cannot reproduce the cognitive, social, and expectancy components of chronic human insomnia. Figure 1 summarizes the proposed framework. It is deliberately organized by evidential distance and testability, rather than by an established causal sequence. The sections below organize mechanisms in three layers: more proximal human network and sleep-physiology evidence; intermediate autonomic, endocrine, and circadian biomarkers; and more distal preclinical hypotheses.

### 4.1 Human network evidence: LC, hypothalamic, circadian, and prefrontal circuits

The most proximal human mechanistic evidence currently comes from network imaging. Sleep–wake transitions depend on reciprocal interactions between arousal-promoting and sleep-promoting systems. The LC–norepinephrine system supports vigilance, attention, and arousal-state switching; its activity changes across wakefulness, NREM sleep, and rapid eye movement sleep (Benarroch, 2018; Eschenko and Sara, 2008). The LC findings of Chen et al. (2023) are biologically compatible with reduced hyperarousal but should be interpreted as putative network-level correlates rather than established causal mediators.

The LC is a small brainstem nucleus, and its functional-connectivity estimates are sensitive to spatial resolution, physiological noise, registration, region-of-interest definition, head motion, scan length, and preprocessing. More generally, small resting-state fMRI samples, multiplicity across regions and edges, scanner or site effects, and analytic flexibility can inflate apparent effects and reduce reproducibility (Chen et al., 2018; Yamashita et al., 2019). Future studies should use preregistered masks and primary contrasts, physiological-noise correction, family-wise error control, intention-to-treat sensitivity analyses, harmonized multisite acquisition, and shared code and masks.

The hypothalamus integrates circadian, homeostatic, endocrine, autonomic, and emotional information. The ventrolateral preoptic area contains sleep-promoting GABAergic and galaninergic neurons, whereas lateral hypothalamic orexinergic circuits promote wakefulness. Direct evidence that acupuncture activates human ventrolateral preoptic neurons is lacking. However, the hypothalamic connectivity findings of Peng et al. (2024) suggest that acupuncture may influence interactions between hypothalamic subregions and prefrontal-orbitofrontal networks involved in affective arousal and sleep-related rumination. SCN connectivity alterations in chronic insomnia disorder further support the relevance of circadian network dysfunction (Yu et al., 2024). These findings define a plausible circuit hypothesis, not proof of hypothalamic normalization.

A broader network interpretation includes the insula, supramarginal gyrus, anterior cingulate cortex, hippocampus, amygdala, dorsolateral prefrontal cortex, salience network, and default-mode network. These regions fit hypotheses about sensory vigilance, cognitive arousal, rumination, and interoception, but apparent regional convergence should not be mistaken for independent replication: Chen et al. (2023) and Jiang et al. (2024) analyzed different endpoints from one registered trial. Resting-state fMRI also cannot by itself separate symptom relief, expectancy, non-specific sensory input, altered body attention, and regression to the mean. Connectivity findings should remain exploratory or associative until independently replicated and shown to temporally mediate objective and subjective sleep change.

### 4.2 Objective sleep physiology and electrophysiological targets

A neuroscience Review of acupuncture for insomnia should move beyond sleep questionnaires and conventional sleep-stage percentages. Insomnia is often associated with cortical hyperarousal, including increased high-frequency EEG activity around sleep onset or during NREM sleep (Perlis et al., 2001; Zhao W. et al., 2021). A Frontiers in Neuroscience study further reported elevated beta activity across nighttime sleep and daytime multiple sleep latency EEG in chronic insomnia, supporting a 24-h cortical hyperarousal model in at least some patients (Shi et al., 2022). These observations make spectral EEG a logical mechanistic endpoint for acupuncture trials.

Among proposed mechanistic endpoints, sleep EEG may provide the closest bridge between subjective improvement and sleep–wake physiology because it can quantify arousal burden, NREM stability, spindle activity, and high-frequency cortical activation without relying solely on self-report.

Beyond band-limited power and conventional staging, slow-oscillation–spindle phase coupling can index temporal coordination between cortical slow oscillations and thalamocortical spindle generation, while sensor- or source-space EEG connectivity can characterize distributed organization during sleep onset and NREM sleep. K-complex morphology, cyclic alternating pattern, stage-transition probabilities, arousal-related entropy or complexity, spindle density and topography, and aperiodic spectral components offer complementary measures of instability (Feige et al., 2013; Ghermezian et al., 2023). None has been validated as an acupuncture-responsive mediator in chronic insomnia disorder. Future trials should prespecify detection algorithms, frequency ranges, artifact rejection, connectivity estimators, and multiplicity control and should establish test–retest reliability before relating change to clinical response.

Recent EEG microstate work has begun to move the field in this direction. Liu et al. (2026) studied chronic insomnia patients before and after a 4-week electroacupuncture course and reported improvements in clinical scales accompanied by changes in selected microstate temporal parameters; exploratory machine-learning models suggested that baseline microstate features may have response-prediction value. The study is useful because it places acupuncture closer to human neurophysiology. It remains hypothesis-generating because the sample was limited and rigorous sham-controlled mediation was not established.

Objective sleep monitoring also helps interpret subjective-objective discrepancy. A patient may report better sleep because sleep continuity improved, because sleep-related threat monitoring decreased, because mood or pain improved, or because the treatment context changed the meaning of nocturnal wakefulness. PSG and actigraphy cannot resolve every mechanism, but they can reduce interpretive ambiguity. For a neuromodulation model, improvements in subjective sleep should converge with at least some objective evidence of altered sleep continuity, arousal burden, or sleep microstructure.

### 4.3 Autonomic, vagal, and endocrine evidence

Autonomic imbalance and stress-axis activity are core components of the insomnia hyperarousal model. Acupuncture may affect autonomic function through somatosensory-autonomic reflexes, vagal afferents, nucleus tractus solitarius pathways, and interoceptive cortical networks (Li et al., 2022). This pathway is especially relevant to auricular stimulation and taVNS. Zhang et al. (2021) reported that taVNS altered medial prefrontal functional connectivity in patients with primary insomnia. More recently, a sham-controlled randomized clinical trial in chronic insomnia disorder reported clinically meaningful PSQI improvement after taVNS compared with sham stimulation across an 8-week treatment period and follow-up (Zhang et al., 2024). These findings strengthen the vagal neuromodulation boundary, but they should be interpreted as evidence for taVNS rather than for needle acupuncture itself. Manual acupuncture, electroacupuncture, TEAS, and vagus nerve stimulation recruit different peripheral receptors, stimulation parameters, and expectation contexts.

The HPA axis is another plausible bridge between insomnia, stress, and acupuncture. Some clinical studies report reduced cortisol or related stress markers after acupuncture, and animal studies suggest modulation of corticotropin-releasing hormone, adrenocorticotropic hormone, and corticosterone pathways (Liu et al., 2021; Zhao H. et al., 2024). A recent meta-analysis of sleep and endocrine outcomes reported favorable signals for PSQI, selected PSG measures, serum melatonin, and salivary cortisol, but also showed substantial heterogeneity, inconsistent salivary melatonin findings, and comparator-dependent ISI results (Hu et al., 2026). These endocrine findings are compatible with a stress-axis mechanism, especially in comorbid insomnia with anxiety or depression. They are not yet proof of mediation.

Endocrine and autonomic markers are easily confounded. Sampling time, recent sleep, medication, menstrual and menopausal status, depression, pain, caffeine use, light exposure, respiration, and acute stress can all shape cortisol, melatonin, and heart rate variability. Future studies should use standardized sampling windows, repeated measures, respiratory monitoring for heart rate variability, and mediation models that test whether endocrine or autonomic change temporally accounts for improvement in insomnia severity and objective sleep.

### 4.4 Neurochemical and neurohormonal hypotheses

GABAergic signaling is central to sleep initiation and suppression of arousal systems. Acupuncture studies have reported changes in GABA-related markers, but the level of evidence varies sharply. In rodent insomnia models, acupuncture has been linked to altered hypothalamic GABA, glutamate, and glutamine cycling, including a lower glutamate/GABA ratio (Xu et al., 2025). The TRN study by Shu et al. (2025) also supports the idea that acupuncture may influence inhibitory thalamic gating. These studies provide mechanistic access but remain indirect for human chronic insomnia.

Human data for GABA mechanisms are less direct. Clinical studies measuring serum GABA can support a physiological association but cannot determine whether central GABAergic synaptic transmission has changed. This point is important because serum neurotransmitter findings are often overstated. The more careful interpretation is that acupuncture may influence inhibitory-excitatory balance, and that this hypothesis now requires magnetic resonance spectroscopy, PSG microstructure, or mechanistic mediation studies in humans.

Serotonin and melatonin findings should be handled similarly. Serotonergic raphe systems and melatonin rhythms are relevant to sleep timing and sleep continuity. Foroughinia et al. (2020) reported increased urinary 6-sulfatoxymelatonin after acupuncture in pregnancy-related insomnia. Animal studies suggest effects on pineal, hypothalamic, and clock-gene pathways, including Clock and Bmal1 expression (Hong et al., 2020; Huang et al., 2023). The evidence is suggestive rather than decisive because few clinical trials measure dim-light melatonin onset, circadian phase, light exposure, or longitudinal rhythm stability.

### 4.5 Preclinical and emerging hypotheses: TRN, gut-brain, and neuroimmune pathways

The TRN is an emerging but still preclinical target. It is rich in GABAergic neurons and participates in thalamocortical gating and sleep spindle generation. Shu et al. (2025) reported that acupuncture modulated neuronal function and synapse-related proteins in the TRN of p-chlorophenylalanine-induced insomnia rats, with involvement of alpha7 nicotinic acetylcholine receptor signaling. This finding is mechanistically interesting, but it should be described as a hypothesis-generating animal result rather than a proven clinical mechanism.

The microbiota-gut-brain axis has been proposed as a pathway relevant to insomnia and acupuncture (Guo et al., 2024). Human case–control studies associate insomnia with differences in microbial diversity and taxa, but heterogeneity in sampling, sequencing, diet, geography, medication exposure, and comorbidity—and the possibility of reverse causation—preclude directional or causal inference. Crucially, no convincing human study has shown that acupuncture-induced microbiome or metabolite change precedes and mediates improvement in objectively measured sleep. The microbiota–gut–brain pathway is therefore retained as a distal, hypothesis-generating route rather than a demonstrated human mechanism.

Acupuncture-specific evidence remains predominantly preclinical. Hong et al. (2020) reported co-occurring sleep-like behavioral and microbiota changes in PCPA-induced sleep-disruption mice, and metagenomic work in PCPA-induced serotonergic-depletion rats reported microbiome and metabolic associations after acupuncture (Qi et al., 2025). These models do not reproduce human chronic insomnia or establish microbial mediation. A decisive program would combine longitudinal randomized human trials, control of diet and microbiome-active medications, shotgun metagenomics and metabolomics, objective sleep outcomes, and—in preclinical causal work—microbiota-transfer or depletion experiments. Table 2 summarizes the causal status and translational distance of the proposed pathways.

**Table 2:** Evidence hierarchy for proposed neurobiological mechanisms.

Pathway	Evidence source	Human-insomnia proximity	Causal status	Main limitation	Best next experiment
Human arousal-network connectivity (LC, hypothalamus, SCN, insula, prefrontal and emotional networks)	Human fMRI in insomnia disorder or CID	High proximity, but samples are small	Associative; not causal	Overlapping cohorts, small single-site samples, ROI and multiplicity choices, motion and physiological noise, scanner/site effects, analytic flexibility, and no mediation.	Harmonized multisite acquisition; preregistered contrasts and masks; family-wise error control; intention-to-treat sensitivity; code sharing; external replication; temporal mediation.
EEG microstructure and thalamocortical gating	Insomnia physiology literature and emerging electroacupuncture EEG microstate work	Moderate for insomnia physiology; early for acupuncture-specific mechanism	Proposed endpoint; not a validated mediator	Sparse sham-controlled spectral and microstructural data; detection algorithms and multiplicity often unspecified.	Mechanistic RCT with prespecified spectral power, SO–spindle coupling, sensor/source connectivity, CAP, transitions, entropy/complexity, reliability, and mediation.
Autonomic-vagal pathways	Human autonomic models; taVNS functional connectivity and randomized clinical evidence; TEAS studies; limited needle-acupuncture data	Low-to-moderate, modality-dependent	Associated or speculative	Manual acupuncture, EA, TEAS, and taVNS are often conflated despite different afferent targets and stimulation parameters.	Separate modality-specific trials with HRV, respiratory measures, skin conductance, actigraphy, credible comparators, and mediation models.
HPA-axis regulation	Human endocrine biomarkers; animal stress-axis studies	Low-to-moderate	Proposed mediator	Sampling time, mood, pain, medication, recent sleep, and light exposure confound endocrine markers.	Repeated standardized cortisol/ACTH sampling with sleep outcomes and stress-arousal mediation testing.
GABA-glutamate balance	Mostly animal studies; occasional peripheral human biomarkers	Low	Mostly preclinical	Serum GABA does not prove central synaptic change; rodent PCPA models differ from human CID.	Human MRS plus PSG microstructure, or pharmacological/physiological mediation designs.
Serotonin and melatonin/circadian signaling	Pregnancy-related clinical biomarker data; animal clock-gene studies; endocrine meta-analysis	Low-to-moderate and population-specific	Associated	Few trials measure dim-light melatonin onset, circadian phase, light exposure, or rhythm stability.	Circadian phenotyping with DLMO, actigraphy, light exposure, and melatonin metabolite trajectories.
TRN and alpha7-nAChR signaling	Animal TRN studies	Very low	Hypothesis-generating	No direct human TRN evidence for acupuncture in insomnia.	Animal EEG-defined sleep plus human spindle/thalamocortical coupling outcomes in translational trial designs.
Gut-brain and neuroimmune signaling	Human case–control associations; predominantly PCPA rodent acupuncture studies	Very low; no human acupuncture mediation	Hypothesis-generating only	Observational human evidence is confounded and directionally uncertain; rodent co-change does not establish microbial mediation of sleep benefit.	Longitudinal randomized human study with diet/medication control, shotgun metagenomics, metabolomics, objective sleep, and temporal mediation; causal transfer/depletion in animals.

Evidence strength reflects human-insomnia proximity and causal interpretability rather than biological plausibility alone. Proximity was judged higher when studies enrolled chronic insomnia disorder or closely related insomnia phenotypes and used human neurophysiology, neuroimaging, or objective sleep outcomes. Causal interpretability was downgraded when evidence came primarily from serum biomarkers, non-insomnia populations, non-needle modalities, or animal models without mediation testing.

## 5 Discussion

The literature has moved beyond a simple question of whether acupuncture improves sleep scores. A recurrent but heterogeneous subjective clinical signal coexists with biologically plausible routes through which peripheral stimulation could influence arousal systems. Evidence remains too uneven to claim targeted regulation of sleep–wake circuitry in a strong causal sense. The main sources of ambiguity are active sham procedures, expectancy, subjective–objective discrepancy, population heterogeneity, modality differences, and the frequent gap between biomarkers and mechanisms.

### 5.1 Sham controls, expectancy, and contextual effects

The first controversy is the sham-control problem. Superficial needling, non-indicated points, non-penetrating devices, and minimal electrical stimulation can all produce sensory input, expectation, relaxation, and physiological effects. Sham-group change also combines expectancy with natural history, regression to the mean, and co-interventions; it is not a pure estimate of placebo. A small real–sham difference may therefore reflect a modest specific effect, an active comparator, or both (Yeung et al., 2018).

From a predictive-processing perspective, sleep perception is an inference formed from noisy interoceptive signals together with prior beliefs, attention, and the precision assigned to sleep-related threat cues (Wager and Atlas, 2015; Ongaro and Kaptchuk, 2019). Treatment ritual, practitioner communication, needling sensation, and beliefs about group assignment may reduce threat monitoring and alter perceived—and in some studies objectively estimated—sleep without engaging an acupuncture-specific pathway (Yeung et al., 2018, 2020). This account is supported most directly by general placebo neuroscience and remains a hypothesis when transferred to acupuncture for insomnia.

Contextual processes can recruit prefrontal, cingulate, insular, autonomic, and subcortical systems that overlap with networks interpreted as neuromodulatory targets. Trials should therefore measure expectancy before treatment, assess intervention credibility and allocation guesses, repeat outcome-expectation ratings during treatment, and test whether expectancy mediates or modifies clinical, EEG, and imaging change rather than treating it only as a nuisance covariate.

### 5.2 Subjective-objective discrepancy in insomnia outcomes

The second problem is outcome selection. PSQI and ISI are clinically meaningful and easy to administer, but they are subjective. They may improve because patients sleep better, worry less about sleep, perceive sleep differently, experience less pain or affective distress, or feel cared for. Objective measures such as PSG and actigraphy are not perfect either: a single laboratory night can miss habitual sleep, and actigraphy estimates sleep from movement. Still, the subjective-objective gap is central to insomnia research.

Recent sham-controlled syntheses illustrate this divide. Yu et al. (2025) reported that subjective PSQI and ISI favored acupuncture, whereas objective total sleep time remained uncertain and the information size for sleep efficiency and wake after sleep onset was limited. This does not invalidate subjective benefit. It means that a mechanistic neuromodulation model must not rest on subjective scales alone. Trials that claim circuit-level modulation should include objective sleep continuity, spectral EEG, arousal index, autonomic indices, or preregistered imaging outcomes that can be tested as mediators.

### 5.3 Population heterogeneity and clinical boundary conditions

The third challenge is heterogeneity. Trials differ in diagnostic criteria, chronicity, comorbidity, acupoint prescription, needling depth, stimulation intensity, de qi requirement, session frequency, total dose, practitioner training, co-interventions, and comparator choice. This heterogeneity is not merely statistical; it reflects different treatment theories. STRICTA-level reporting is therefore essential for reproducibility and for identifying whether dose, acupoint selection, or stimulation parameters matter (MacPherson et al., 2010).

Population mixing is a related problem. Chronic insomnia disorder, depression-related insomnia, post-stroke insomnia, pregnancy-related insomnia, cancer survivor insomnia, pain-related insomnia, and perioperative sleep disturbance differ in mechanisms, risks, goals, and acceptable comparators. Combining these populations without explanation can make acupuncture appear more broadly established than the evidence warrants. Table 1 instead assigns narrative confidence to the specific inference shown; clinical benefit and mechanistic interpretation may receive different confidence within the same study.

### 5.4 Modality specificity: acupuncture, electroacupuncture, TEAS, and taVNS are not the same intervention

A recurring weakness in the literature is the tendency to collapse different peripheral stimulation methods into a single category. Manual acupuncture, electroacupuncture, TEAS, auricular stimulation, acupressure, and taVNS differ in tissue penetration, receptor recruitment, stimulation frequency, current intensity, practitioner interaction, and patient expectation. A mechanism inferred from one modality should not be automatically assigned to another.

For example, taVNS findings are relevant to vagal-interoceptive models, but they do not prove that manual acupuncture modulates vagal tone in the same way. Electroacupuncture permits more standardized stimulus parameters than manual needling, but it also introduces device-specific and electrical-stimulation effects. Future studies should treat modality as an experimental factor, not a nuisance variable. This is especially important if acupuncture is framed as peripheral neuromodulation rather than as a generic complementary therapy.

### 5.5 Mechanistic overreach and causal inference

The final scientific challenge is mechanistic overreach. It is tempting to write that acupuncture normalizes GABA, restores the HPA axis, rewires brain networks, or acts through gut microbiota. In most cases, the data do not support such certainty. More accurate wording is modest: acupuncture may influence, is associated with, or provides a plausible route through a pathway. This distinction is substantive because it separates an accompanying signal from a causal mediator.

A stronger mechanistic field would test mediation. If LC connectivity, hypothalamic-prefrontal coupling, HPA-axis downregulation, vagal activation, EEG microstructure, or GABA-glutamate balance is proposed as a mechanism, the relevant measure should be collected before, during, and after treatment and tested statistically as a mediator of clinical improvement. Without this step, biomarkers remain accompanying signals, not mechanisms. Accordingly, the minimum evidentiary criteria for evaluating acupuncture-related stimulation as a neuromodulatory approach to insomnia are summarized in Box 1.

Box 1 **Minimum evidentiary criteria for treating acupuncture-related stimulation as a neuromodulatory model of insomnia**

• Clinical improvement should exceed a credible sham comparison or have a clearly defined relationship to an active comparator such as CBT-I.
•Subjective improvement should converge with objective sleep measures, such as PSG, actigraphy, spectral EEG, arousal index, or sleep microstructure.
•A proposed biomarker should change before, or at least in parallel with, clinical improvement and should show temporal plausibility.
•Mechanistic measures should be tested as mediators rather than described only as accompanying correlates.
•Expectancy, treatment preference, therapeutic attention, and blinding credibility should be measured and modeled.
•Animal or serum-marker findings should not be directly extrapolated to human central sleep-wake circuitry without intermediate human evidence.

### 5.6 Evidence that would weaken the neuromodulation model

A useful model should specify what findings would weaken it. The peripheral neuromodulation interpretation would be substantially downgraded if adequately powered, sham-controlled trials repeatedly showed improvement in PSQI or ISI without convergent change in objective sleep continuity or architecture, actigraphy, spectral EEG, heart rate variability, endocrine rhythm, or preregistered LC, hypothalamic, or thalamocortical connectivity measures. It would also be weakened if expectancy and blinding credibility fully explained clinical improvement, or if mechanistic changes occurred without temporal precedence or statistical mediation of sleep outcomes.

Such findings would not prove that acupuncture has no clinical value. They would instead shift the most parsimonious interpretation toward nonspecific sensory stimulation, contextual care, expectancy, relaxation, or altered sleep perception rather than targeted modulation of sleep-wake circuits. Stating these disconfirmation conditions makes the field more falsifiable and more aligned with translational neuroscience.

### 5.7 Safety, precautions, and implementation boundaries

Large prospective data across broad indications—not insomnia-specific incidence estimates—suggest that acupuncture delivered by trained practitioners is generally associated with minor, self-limited events. A meta-analysis of prospective studies estimated that 9.31% of patients experienced at least one adverse event; transient bleeding, needling pain, and local symptom flare accounted for about half of events, while estimates varied substantially with definitions and surveillance (Bäumler et al., 2021). Pneumothorax, infection, retained or broken needles, and tissue or nerve injury are uncommon but clinically important. Risk is modified by anatomical site and depth, practitioner training, bleeding tendency, frailty, impaired immunity, and local skin disease; pregnancy and anticoagulation warrant individualized assessment rather than blanket exclusion.

Safety is modality-specific. Electroacupuncture and TEAS studies should report current, pulse width, electrode or needle placement, skin reactions, and precautions related to implanted electronic devices. For taVNS, a systematic review found no greater overall adverse-event risk than control stimulation, with ear pain, headache, and tingling among the most frequent events; however, more than half of included studies did not state whether adverse events occurred (Kim et al., 2022). Trials should report denominators, severity, relatedness, withdrawals, and serious events separately for each modality.

### 5.8 Economic evidence and implementation

Economic evidence is substantially less mature than efficacy evidence, and portable cost comparisons cannot be inferred from session or drug list prices across health systems. Reviews of insomnia interventions find the most consistent cost-effectiveness evidence for CBT-I, including digital delivery, while complementary and alternative interventions remain under-researched (Fang et al., 2024; Le et al., 2026). A recent South Korean model suggested that pharmacopuncture could be cost-effective relative to conventional acupuncture under local willingness-to-pay assumptions, but the result was price-sensitive and pharmacopuncture combines needling with injected herbal products (Cho et al., 2026). It neither compared standard acupuncture with CBT-I or pharmacotherapy nor establishes generalizability beyond that setting.

Future economic evaluations should be jurisdiction-specific and prospectively collect practitioner and facility time, training and device costs, number of sessions, CBT-I delivery mode, medication and adverse-event costs, patient out-of-pocket expenses, adherence, retreatment and relapse, downstream health-care use, productivity loss, insomnia remission, and quality-adjusted life-years. Trial-based analyses should report health-system and societal perspectives, extend beyond the immediate treatment period, and include deterministic and probabilistic sensitivity analyses. Until such data are available, acupuncture is better described as a potentially accessible adjunct in some settings than as a proven lower-cost alternative.

### 5.9 Translational implications and future directions

Patients should be told that certainty varies by condition and outcome. For chronic insomnia disorder, the best-supported outcomes are subjective sleep quality and insomnia severity. For cancer survivors, CBT-I remains the stronger benchmark for insomnia severity immediately after treatment, while acupuncture may be useful when pain and other somatic symptoms are prominent (Garland et al., 2019; Yang et al., 2021). For post-stroke insomnia or depression-related insomnia, acupuncture may be considered within multidisciplinary care rather than as a stand-alone sleep intervention.

Future trials should be designed around clearly separable questions: superiority over sham, non-inferiority or add-on value relative to CBT-I, relapse prevention, and mechanism testing. These questions require different comparators, sample sizes, outcomes, and follow-up periods. Preregistration, concealed allocation, assessor blinding, expectancy assessment, STRICTA-compliant intervention reporting, and transparent adverse-event reporting should be standard.

For mechanistic trials, objective sleep physiology should be part of the primary design rather than an exploratory add-on. The most informative designs would combine credible sham or active comparators, PSG and multiday actigraphy, spectral EEG or sleep microstructure, autonomic-endocrine sampling, preregistered imaging targets, expectancy assessment, and mediation models. The next step is not simply more small positive trials; it is randomized mechanistic work that can separate causal pathways from correlates of clinical improvement.

Preclinical work should also better match clinical phenotypes. PCPA models are useful for serotonergic depletion but do not capture all aspects of chronic insomnia disorder. Stress-induced, circadian-disruption, pain-related, and mood-related models may be more appropriate for different patient subgroups. Translation would improve if animal studies included EEG-defined sleep stages, autonomic measures, and behavioral readouts of hyperarousal.

### 5.10 Limitations of this review

This Review has limitations. It is a purposive narrative synthesis, not a systematic review, and did not independently screen all Chinese-language databases, Embase, or Web of Science with duplicate reviewers. The 360-record PubMed count is a reproducibility audit of the stated core string, whereas earlier iterative supplementation was not prospectively logged and cannot support a conventional PRISMA eligibility flow. Publication bias, language bias, selective outcome reporting, and the concentration of trials in single-country settings may influence the evidence discussed. The Review also emphasizes mechanistically informative sources rather than every available clinical trial; it should therefore be read as a critical translational synthesis, not a definitive treatment guideline.

A second limitation is that mechanistic interpretation depends on the quality of the underlying studies. Many trials have small samples, short follow-up, imperfect sham controls, limited objective sleep measurement, and no formal mediation analysis. Many animal studies use models that do not fully capture chronic human insomnia. These constraints do not make the field unscientific, but they should keep the claims modest.

## 6 Conclusion

Acupuncture-related peripheral stimulation for insomnia has moved from broad clinical observation toward a more mechanistically informed field. Current evidence supports an outcome-dependent signal for improvements in subjective sleep quality and insomnia severity in defined clinical contexts. Evidence for objective sleep architecture, long-term maintenance, and biomarker-mediated clinical response remains insufficiently established.

Human network and physiology studies identify LC and hypothalamic connectivity, emotional and interoceptive networks, EEG hyperarousal and microstructure, autonomic–vagal function, HPA-axis activity, circadian markers, and thalamocortical gating as testable targets. Preclinical work adds GABA–glutamate, TRN, neuroimmune, and microbiota–gut–brain hypotheses, but these pathways remain at different translational distances from human chronic insomnia disorder. The most defensible position is a setting-dependent, adjunctive, preference-sensitive, and phenotype-aware proposed neuromodulatory model until rigorous trials link clinical response to objective sleep physiology and prespecified mediators. Safety appears generally favorable with trained delivery but requires modality-specific reporting, and comparative cost-effectiveness against CBT-I or pharmacotherapy has not been established.
