# Supplementary material for: Acupuncture-related peripheral stimulation as a candidate neuromodulatory model of insomnia: clinical evidence, sleep-wake circuitry, and translational limits
Source: Front Neurosci. 2026 Aug 26;20:1884710. doi: 10.3389/fnins.2026.1884710 (PMC13557252; doi:10.3389/fnins.2026.1884710)
Supplement: Supplementary file 1 [file Table_1.docx]

**Supplementary Methods S1. Literature search and narrative appraisal procedures**

**Review type and purpose**

The Review used purposive source selection to integrate representative clinical, imaging, physiological, biomarker, safety, economic, and preclinical evidence. Its purpose was conceptual synthesis rather than a pooled efficacy estimate. Duplicate screening, formal study-level risk-of-bias grading, and PRISMA-based evidence synthesis were not performed.

**Sources and dates**

The exact core PubMed query was rerun on 7 August 2026. Targeted searches of the Cochrane Library, publisher pages, reference lists, and major evidence syntheses were completed through 11 May 2026 and updated selectively during revision. Reviews that searched CNKI, Wanfang, VIP, or SinoMed were used as evidence maps, not as a substitute for an independent bilingual review.

**Exact core PubMed query**

(insomnia disorder OR chronic insomnia OR primary insomnia) AND (acupuncture OR electroacupuncture OR transcutaneous electrical acupoint stimulation OR auricular stimulation OR acupoint stimulation) AND (sham acupuncture OR polysomnography OR actigraphy OR electroencephalography OR spectral EEG OR fMRI OR functional connectivity OR locus coeruleus OR hypothalamus OR suprachiasmatic nucleus OR thalamic reticular nucleus OR HPA axis OR GABA OR glutamate OR serotonin OR melatonin OR heart rate variability OR microbiota OR gut-brain axis)

**Title-level mapping and unavailable counts**

The query returned 360 records. All titles were reviewed once during revision and assigned to one of five prioritization categories: 92 direct chronic-insomnia or human acupuncture-related, 82 indirect clinical or acute-state, 122 human sleep-mechanism, review, or guideline, 20 preclinical insomnia, and 44 clearly irrelevant records. These were not full-text eligibility decisions. Record-level assignments are supplied in Supplementary Data S1.

Earlier supplementary searches were iterative, and unique records were not prospectively retained. Deduplicated and full-text counts are therefore unavailable. The 63 cited publications draw on the core search and targeted supplementation and are not a PRISMA-defined included-study set.

**Appraisal framework**

Evidence was appraised by design, directness to chronic insomnia disorder, objective measurement, replication, and causal interpretability. Human imaging, physiological, autonomic, endocrine, and biomarker findings were treated as associative unless temporal mediation was tested; animal and cellular findings were preclinical or hypothesis-generating. Narrative confidence applies to the stated inference and is not a formal GRADE rating. Definitions are provided in Supplementary Table S2.

**Supplementary Figure S1. Narrative source-selection audit trail**

*Supplementary Figure S1. Narrative source-selection audit trail. The exact core PubMed query returned 360 records on 7 August 2026. Of the 360 titles reviewed once during revision, 316 were potentially relevant and 44 were clearly irrelevant; the five-category breakdown is reported in Supplementary Table S1 and the record-level assignments are supplied in Supplementary Data S1. These were prioritization categories, not full-text eligibility decisions. Earlier deduplicated and full-text counts were not prospectively retained. The final Review cites 63 publications from the core search and targeted supplementation. This figure is not a PRISMA eligibility flow.*

**Supplementary Table S1. Search-source audit**

The table separates directly auditable counts from iterative source-finding activities for which unique counts were not prospectively retained.

| **Source** | **Date** | **Strategy or audit action** | **Verified count** | **Role and limitation** |
| --- | --- | --- | --- | --- |
| **PubMed** | 7 August 2026 | Exact three-concept core query reproduced in Supplementary Methods S1. Title metadata were exported and mapped once using categories defined for the revision-stage audit. | 360 records and titles mapped: 92 direct CID or human acupuncture-related; 82 indirect clinical or acute state; 122 human mechanism, review, or guideline; 20 preclinical insomnia; 44 clearly irrelevant. | Core clinical, human mechanistic, biomarker, review, guideline, and preclinical sources. Single title-level mapping; not duplicate screening or full-text eligibility review. |
| **Cochrane Library** | Through 11 May 2026 | Targeted combinations of insomnia terms with acupuncture, electroacupuncture, acupoint stimulation, sham, and objective-sleep terms. Exact platform syntax and a unique record log were not retained. | Unique record count unavailable. | Used to identify evidence syntheses and controlled trials. Deduplicated and full-text counts cannot be reconstructed. |
| **Publisher pages and reference lists** | Through 11 May 2026; selective updates during revision | Trial names, authors, modalities, mechanisms, DOI checks, backward citation review, and targeted evidence updates. | Iterative; unique count unavailable. | Verification and supplementation. Not pooled with PubMed because record-level logs were not retained. |
| **Chinese-language evidence maps** | Evidence syntheses available through revision | Recent systematic reviews cited in the Review that searched one or more of CNKI, Wanfang, VIP, and SinoMed were used as evidence maps; no independent bilingual database screen was claimed. | Not applicable to this audit. | Broadens contextual coverage but cannot replace an independent bilingual systematic review. |
| **Final cited synthesis** | 7 August 2026 | Critical appraisal and conceptual integration across the core query and targeted supplementation. | 63 publications cited. | Final cited sources, not a PRISMA-defined eligible-study set and not all derived from the 360-record PubMed result. |

*Note. CID, chronic insomnia disorder. The five PubMed categories were used for title-level prioritization and were not eligibility decisions. The complete record-level audit is provided in Supplementary Data S1.*

**Abbreviations**

CBT-I, cognitive behavioral therapy for insomnia; CID, chronic insomnia disorder; EEG, electroencephalography; fMRI, functional magnetic resonance imaging; GRADE, Grading of Recommendations Assessment, Development and Evaluation; HPA, hypothalamic-pituitary-adrenal; ISI, Insomnia Severity Index; LC, locus coeruleus; PSG, polysomnography; PSQI, Pittsburgh Sleep Quality Index; SCN, suprachiasmatic nucleus; TEAS, transcutaneous electrical acupoint stimulation; TRN, thalamic reticular nucleus; taVNS, transcutaneous auricular vagus nerve stimulation.

**Alt text for Supplementary Figure S1**

*Narrative source-selection audit trail. The core PubMed query returned 360 records on 7 August 2026, and all 360 titles were mapped once during revision. Three hundred sixteen titles were potentially relevant and 44 were clearly irrelevant. Iterative targeted supplementation had no retained unique count. The final narrative synthesis cited 63 publications. The diagram is not a PRISMA eligibility flow.*

**Supplementary Table S2. Evidence-appraisal definitions**

These definitions were used to keep clinical proximity, causal interpretation, and confidence in a stated inference conceptually separate.

| **Domain** | **Label** | **Operational definition** | **Permitted interpretation** |
| --- | --- | --- | --- |
| Title-level mapping | **A. Direct CID or human acupuncture-related** | The title directly concerned chronic insomnia disorder, or a human clinical or mechanistic study of an acupuncture-related intervention with a sleep-relevant outcome. | Prioritized for direct clinical or modality-specific evidence; not a full-text eligibility decision. |
| Title-level mapping | **B. Indirect clinical or acute state** | The title concerned a comorbid population, special population, perioperative or acute sleep disturbance, or a non-insomnia condition in which sleep was secondary. | Used only to extend or bound the clinical rationale; not treated as interchangeable with CID. |
| Title-level mapping | **C. Human sleep mechanism, review, or guideline** | The title concerned human sleep physiology, neuroimaging, biomarkers, insomnia reviews, evidence syntheses, or clinical guidance without direct acupuncture-specific CID evidence. | Used for mechanism, context, comparator, or standard-of-care framing. |
| Title-level mapping | **D. Preclinical insomnia** | The title concerned animal, cellular, or other preclinical models of insomnia or sleep disruption with a mechanism relevant to the Review. | Treated as preclinical or hypothesis-generating and not as proof of a human mechanism. |
| Title-level mapping | **E. Clearly irrelevant** | The title did not address a sleep-relevant clinical, mechanistic, review, guideline, or preclinical question within the Review scope. | Not prioritized for further narrative appraisal. |
| Clinical proximity | **High** | Human randomized, sham-controlled, physiological, or imaging evidence in chronic insomnia disorder. | Can support a direct clinical or associative human inference, depending on design; mechanism still requires mediation. |
| Clinical proximity | **Moderate** | Human evidence in comorbid insomnia, special populations, or acute sleep disturbance, or indirect human sleep physiology relevant to the pathway. | Can support contextual or pathway plausibility but not direct generalization to CID. |
| Clinical proximity | **Low or very low** | Predominantly peripheral biomarkers, non-insomnia models, animal studies, or cellular work. | Can generate hypotheses but cannot establish a human CID mechanism. |
| Causal status | **Associative human evidence** | A clinical, imaging, physiological, autonomic, endocrine, or biomarker change accompanied or correlated with symptom change without temporal mediation. | May refine a mechanism hypothesis; does not identify a treatment-specific mediator. |
| Causal status | **Proposed mediator** | A biologically plausible intermediate outcome has been identified, but temporal precedence and formal mediation have not been established. | Should be described as a candidate endpoint or proposed mediator, not a demonstrated mechanism. |
| Causal status | **Preclinical or hypothesis-generating** | Evidence is derived mainly from animal or cellular intervention models, or from distal human associations without acupuncture-specific mediation. | Supports a testable route only; human causal interpretation is not warranted. |
| Narrative confidence | **Higher / moderate / low or very low** | Higher requires direct design, objective measurement, reasonable precision, consistency, replication, and limited causal ambiguity. Moderate retains one or more important limitations. Low or very low denotes indirect, imprecise, heterogeneous, unreplicated, associative, or preclinical evidence. | Applies only to the stated inference and supports language calibrated to the remaining uncertainty; it is not a global study grade. |

*Note. Confidence labels are a structured narrative appraisal of the specific inference shown. They are not formal GRADE ratings or global study-quality scores.*
